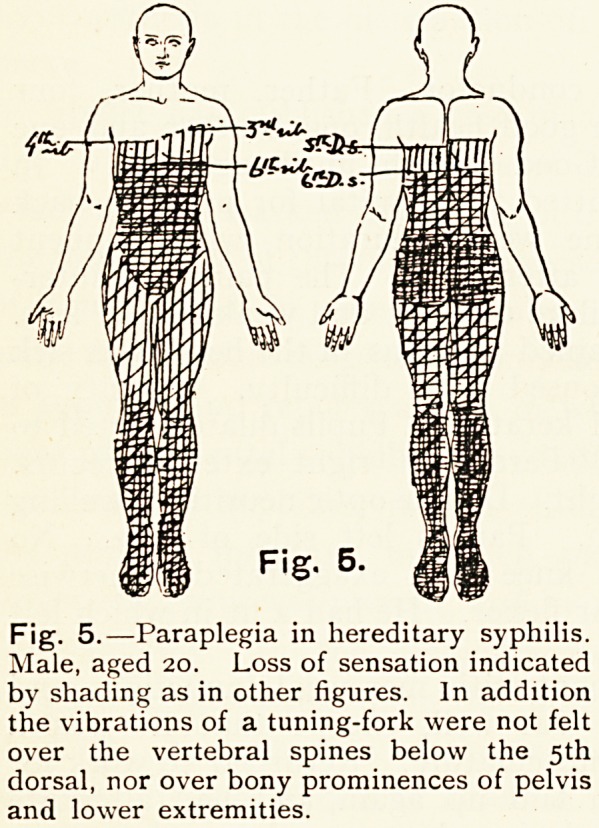# The Long Fox Lecture: Cerebro-Spinal Syphilis

**Published:** 1911-03

**Authors:** J. Michell Clarke

**Affiliations:** Professor of Medicine, University of Bristol; Physician to the Bristol General Hospital


*"Scire est nescire, nisi id me*
*Scire alius sciret."*

MARCH, 1911.

### THE LONG FOX LECTURE:

THE SEVENTH ANNUAL LECTURE ARRANGED BY THE COMMITTEE OF THE LONG FOX MEMORIAL,
DELIVERED IN THE MEDICAL LIBRARY OF THE UNIVERSITY OF BRISTOL ON DECEMBER 15TH, 1910.

THE VICE-CHANCELLOR *in the Chair.*

BY

J. MICHELL CLARKE, M.A., M.D. Cantab., F.R.C.P.,
*Professor of Medicine, University of Bristol; Physician to the Bristol General Hospital.*

ON

### CEREBRO-SPINAL SYPHILIS.

---

BEFORE proceeding to the subject of my lecture, I wish to pay my meed of tribute to the memory of Edward Long Fox, in whose honour these lectures have been instituted. There will come a time when the lecturer of the day will not have known him personally, hence it is the more a pious charge on those who have had the advantage of his friendship to commemorate his virtues. For they know well that Edward Long Fox was a man held in especial honour and affection by his medical brethren in this city and neighbourhood. Honoured for his wide culture and knowledge of his profession, and beloved for

2

his warmth of heart and kindness of disposition, there was no member of his profession to whom in case of need he was not always willing to lend a helping hand, either by wise advise or by substantial aid. I myself am indebted to him on more than one occasion for valuable counsel. Throughout a long professional life he never flagged to the end in maintaining a keen interest in the scientific side of medicine and in its progress. At the same time he followed the best traditions of the profession—long may they flourish amongst us—in keeping in touch with the great matters of literature and knowledge, and he was a man of deep and genuine religious convictions.

In his professional work Edward Long Fox tended especially to the study of neurology, in which he always maintained the greatest interest, at which he was a life-long worker, and attained to eminence in it. Indeed, his book on the *Pathology of the Nervous Centres* marks him as a pioneer in the early days of the long series of pathological investigations which in our time have led to such fruitful results.

In choosing Cerebro-Spinal Syphilis as the subject of this lecture, I therefore feel that it is one in which he himself was greatly interested, and by which I may, in some humble measure, do honour to his memory.

A fundamental fact in pathology is that there can be no accurate knowledge of a disease until the cause is known, nor can that knowledge be full and complete until the disease can be reproduced experimentally. The anatomical, coarse and microscopical, lesions of syphilis have long been known, and the treatment empirically determined. The constant and characteristic histological structure of syphilitic lesions pointed to a reaction on the part of the tissues of the invaded organism to a specific agent. The marked and peculiar infectivity of the disease further indicated the action of a living micro-organism. The rapid advance in bacteriological science, and the discovery that the lesions of tuberculosis, in which again characteristic histological tissue changes are constantly present, are due to a bacillus, naturally led, but fruitlessly, to the search for a bacillus in syphilis. It was from the first, however, recognised by most observers that the course of syphilitic affections was a peculiar one, and did not exactly correspond to that of any known bacterial disease.

In recent times three most important discoveries have been made, by which the study of syphilis has been enormously advanced, and features of the disease, which for centuries remained obscure and impossible of explanation, have been already largely elucidated. These three discoveries are (1) that of the spirochæta pallida or treponema pallida by Schaudinn, and its establishment as the infective agent and cause of syphilis; (2) of certain specific reactions in the blood and cerebro-spinal fluid of the subjects of present or past syphilis, which have already proved of the greatest clinical value in diagnosis; and (3) the experimental communication of the disease to the higher apes.

There is little reason to doubt that the gap in the present knowledge of the life-history of the spirochæta pallida, that is the mode in which it exists outside the body, will shortly be discovered. When this step is accomplished there is equally little reason to doubt that more effective measures for prevention of the disease will be attained, and also further developments in the cure of syphilis in its early stages. The latter have, indeed, if the hopes based upon Ehrlich's new remedy are well-founded, already been successfully started.

The spirochæta pallida is present in all cases of syphilis, and is not found in any other disease. It has been found in every kind of syphilitic lesion, in the changes (reaction by tissues) provoked by the organisms. Mott states that Levaditi's experiments suggest that they stimulate the fixed tissue cells to proliferate, and then invading this bed of young cells, rich in nuclein, the organisms by the action of some secretion or in some other way cause these young cells to undergo lysis, thus providing the necessary pabulum for their own growth and multiplication. Probably these young cells are more easily attacked and destroyed than the older ones, and this may be the reason why the spirochætes are found in such numbers in the fœtal tissues in congenital syphilis.[1] From the seat of primary infection the spirochætes soon reach the nearest lymphatic glands, where they multiply in the sinuses and spaces, and then pass into the general lymph stream, infect distant glands, and by way of the thoracic duct may cause a general infection. The spirochætes have not been found in the cerebro-spinal fluid except in a few instances. [2] Hoffman has, however, succeeded in inoculating an ape with syphilis with cerebro-spinal fluid obtained free from blood from a man with a papular syphilide.

It has been already stated that the course and manifestations of syphilis do not correspond with those of diseases produced by bacteria. Mott [3] has pointed out the great similarity of the histological lesions in the nervous tissues in chronic trypanosome infections, *e.g.* sleeping sickness, and dourine to syphilitic and parasyphilitic lesions. The universal perivascular infiltration in the central nervous system of lymphocytes and plasma cells, formerly thought to be pathognomonic of general paralysis and syphilis, he has shown to be equally characteristic of, and identical in, sleeping sickness, and gives the following points of resemblance and difference between sleeping sickness and syphilis. [4] They resemble one another in (1) early phenomena of infection ; (2) a sore or other irritation at points of entry ; (3) period of incubation ; (4) invasion of local lymphatic glands leading to a general infection of the blood stream ; (5) presence of erythematous rashes with constitutional disturbances due to migration of spirochætes into blood stream ; (6) the occurrence of polyadenitis and lymphocytosis. Points of difference are :—

| SYPHILIS. | SLEEPING SICKNESS. |
| --- | --- |
| Invasion of nervous system in 5 to 10 per cent. only. When spirochætes invade subarachnoid space they remain within lymphatics, and do not become free in cerebro-spinal fluid. They are not found in blood, and multiply only in lymph-spaces and channels. | Chronic inflammatory affection of lymphatics of central nervous system.<br>Subarachnoid space always invaded and trypanosomes free in cerebro-spinal fluid, and usually in blood. |

Mott suggests that the extreme activity of the trypanosomes of sleeping sickness enables them to penetrate the walls of the capillaries, whilst the very slow and screw-like movements of the spirochæta pallida do not allow it to do so, and also that the deadly results of the *Trypanosoma gambiense* are due to the organism having acquired the power of passing into the subarachnoid space.

Passing over the second point, the communication experimentally of syphilis to the higher apes, the third discovery is that of Wassermann's and of other tests which in competent hands have already proved so valuable in diagnosis. For details as to these tests the reader may be referred to Mott's admirable Morison Lectures, from which I have already largely quoted. A few words may be said as to the nature of the test in syphilis, and first may be mentioned the fat-like substances known as lipoid bodies contained in all living cells, and probably essential to their vital activity. They consist of (1) nitrogen and phosphorus free bodies, cholesterol-fatty acids and lipochromes; (2) phosphorus free, but containing nitrogen, cerebrosides; (3) nitrogen and phosphorus containing phosphatides, lecithin, myelin. Munk[5] showed that the presence of lipoid droplets in a cell indicated dissolution of the nucleus and destruction of the cell, so that lipoid substances in great abundance indicate the breaking up of the nucleus, which contains much phosphorus, and the cell-protoplasm into lipoid substances.

Thus in diseases attended with the degeneration of neurones, *e.g.* tabes, general paralysis, thrombotic softening, etc., the destruction of the ganglion cells and neurones generally leads to the constant presence in the cerebro-spinal fluid of cytolytic products, lipoids, phosphatides and cholestol, globulin and nucleo-protein. These products act as irritants, and excite a lymphocytosis in the cerebro-spinal fluid, cell-infiltrations, and changes in the ependyma of the ventricle. This lymphocytosis is most marked in the parasyphilitic affections in which also the above substances are found in greatest abundance. Although it has now been shown that the syphilitic virus induces metabolic changes, whereby larger amounts of lipoid substances are present in the serum and cerebro-spinal fluid in general paralysis and tabes than in other diseases, yet these substances also exist in normal serums, and the change is therefore rather a quantitative than a qualitative one.

Levaditi and Yamanouchi state that in Wassermann's reaction the use of extracts of liver as antigen depends on the presence of bile salts and (lecithin-containing) lipoids, and certain substances in the serum which are not syphilitic antibodies, but bind up with certain constituents of normal and syphilitic tissues. The serum reaction of syphilis and general paralysis is the same, and has no relation to spirochæta pallida, and is not due to the intervention of syphilitic antibody or antigen in the usual sense of these words.

They consider that Wassermann's reaction is due to the presence in the serum and cerebro-spinal fluid of unknown bodies, which may be cholesterol-esters, which precipitate and determine the fixation of the complement in presence of bile salts, soaps, lecithin, etc. Wassermann's reaction is caused by a histogenic and not a bacterial substance, and is quantitative and not qualitative.

These new views do not militate against the clinical importance of the test. Mott thinks it probable that the syphilitic virus brings about an increased loosening of the complex lipoids containing lecithin and cholesterin from the cells of the body and the red corpuscles of the blood throughout life. In tabes and general paralysis this affects the central nervous system, which normally is protected against the loss of its lipoid substances, and hence their presence in the cerebro-spinal fluid.

To conclude, Wassermann's reaction is positive in nearly all cases of syphilis, is a specific reaction, but does not indicate the organ affected. It may be obtained after twelve to fourteen days in primary syphilis. As it is a complicated process, difficult to carry out, efforts have been made to simplify it. The most promising modification seems to be Fleming's test.[6] This depends upon the fact that human serum is normally hæmolytic to the red corpuscles of the sheep. The three bodies required for this test are therefore the antigen (extract of liver or heart muscle), the suspected serum, and washed sheep's corpuscles.

Noguchi's test depends upon the presence of a globulin in the blood serum and cerebro-spinal fluid in syphilis. The slightest trace of blood in the latter renders it valueless for this test. This globulin has been identified with euglobulin. Noguchi states "that the high value in complement binding exhibited by blood serum from syphilitics and by spinal fluids from general paralysis is associated with an excessively high content of globulin, but there does not exist a direct quantitative relation between the two."[7]

We may now turn to the results obtained by the application of Wassermann's, Noguchi's and Fleming's tests in the diagnosis of cerebro-spinal syphilis and parasyphilitic affections. I am much indebted to Mr. Scott Williamson, Pathologist to the Bristol General Hospital, for kindly giving me the results of his researches into the comparative value of the different tests. Mr. Williamson considers that Wassermann's reaction with careful technique is a quantitative method, and that Noguchi's modification falls into the same category. A negative result from Fleming's and Sabrazé's methods always went with a negative Wassermann, but with positive result from the two former seven cases failed to give a positive result by Wassermann used quantitatively. It would therefore seem that a negative Fleming or Sabrazé is more reliable than a positive.

Wassermann's and Fleming's reactions behave similarly under mercurial treatment. The reaction may disappear in a varying time—on an average five to thirteen months in Mr. Williamson's cases. It may not disappear under the most energetic administration of mercury.

The accompanying table gives the chief results.

Other tests of much value in syphilis and parasyphilis of the central nervous system are the presence of a lymphocytosis, and of globulins in the cerebro-spinal fluid. Lymphocytosis is most marked in parasyphilitic affections, namely tabes and general paralysis.

Nonne and Holzmann[8] conclude from a study of nearly 300 cases that (1) in tabes and general paralysis, lymphocytosis and globulin reaction are present almost without exception,

TABLE I. WASSERMANN'S REACTION.

| | General Paralysis. | | | Tabes Dorsalis. | | | Cerebro-Spinal Syphilis. | | |
|---|---|---|---|---|---|---|---|---|---|
| | No. of Cases. | Serum. | C.S. Fluid. | No. of Cases. | Serum. | C.S. Fluid. | No. of Cases. | Serum. | C.S. Fluid. |
| Boas .. .. .. .. .. | — | 94 % + ve | — | — | 78 % + ve | — | — | — | — |
| Ledermann, 1909 .. | 48 | 45 + ve | — | 116 | 110 + ve | — | 45 | 34 + ve | — |
| *Ibid.*, 1910 .. | 23 | 20 + ve | — | 68 | 52 + ve | — | 27 | 24 + ve | 3 + ve |
| Plaut .. .. .. .. .. | 95 | 95 + ve | 94% | — | — | 70–80 % | — | — | — |
| Smith and Candler .. | 64 | 64 + ve | 59 + ve | — | — | — | — | — | — |
| Flashman and Butler .. | — | 98 % + ve | — | — | — | — | — | — | — |
| Mott .. .. .. .. .. | 46 | 46 + ve | 41 + ve | — | — | 60 % | — | — | — |
| Eichelberg .. .. .. .. | 110 | 97 + ve | 93 % | 49 | — | 27 % | 18 | — | 7 + ve |
| Nonne and Holzmann | Total Cases 295* | 100 % + ve | almost 100 % | — | 60–70 % | nearly always – ve, or reaction feeble | | | — |

* Lymphocytosis and globulin reaction + ve in general paralysis and tabes, almost without exception, and seldom fail in cerebro-spinal syphilis.

MR. SCOTT WILLIAMSON'S CASES.

| | Cases. | Wassermann. | Noguchi. | Fleming. | Sabrazé. |
|---|---|---|---|---|---|
| General Paralysis .. .. | 43 | + + | + + | | + |
| Tabes .. .. .. .. .. | 11 | 9 | 9 | 11 | 10 |
| Cerebro-spinal Syphilis .. | 5 | + | + | + | + |
| Gumma Cerebri .. .. | 2 | + | + | + | + |
| Imbecility (C.S. fluid) .. | 13 | 11 | 11 | 13 | 13 |
| Syphilis .. .. .. .. | 37 | 34 | 34 | 37 | 37 |
| Optic Atrophy .. .. | 5 | + | + | + | + |

including incipient cases. (2) Both only seldom fail in cerebro-spinal syphilis. (3) Their results with Wassermann's reaction are given in Table I. They lay stress upon the constant presence of a marked reaction in the cerebro-spinal fluid of general paralysis, and its absence or feebleness in tabes as a distinction between the two diseases. (4) If this test is positive in epilepsy or tumor cerebri, the epilepsy is a sign of cerebral syphilis, and the tumour is a syphilitic neoplasm, or the patient is suffering from syphilis of some other organ. (5) Lymphocytosis and the globulin reaction are not causally related to Wassermann's reaction in the cerebro-spinal fluid. In syphilitic disease of the nervous system all four reactions, *e.g.* lymphocytosis, globulin-reaction and Wassermann's reaction for serum and cerebro-spinal fluid, are in their intensity and relation to each other independent of the stage and course of the disease.

Eichelberg states that there is no regular correspondence between the Wassermann and globulin reactions. The latter is more often positive than the former in early cases of general paralysis, tabes and cerebro-spinal syphilis.

Further points made by various observers are that the Wassermann reaction is never positive in the cerebro-spinal fluid unless it is positive for the serum, that a negative result in the serum renders general paralysis unlikely, and that in syphilitic patients with positive serum reaction and a normal central nervous system the cerebro-spinal fluid is negative. The test is as a rule negative for the cerebro-spinal fluid in cases of cerebro-spinal syphilis where the serum is positive. A positive Wassermann reaction does not necessarily accompany an excess of lymphocytes. In initial and stationary cases of general paralysis and in old cases of tabes a positive result is not obtained so constantly in the cerebro-spinal fluid as in the serum. Zaloziecki holds that in a very early stage of general paralysis a negative result does not absolutely exclude the disease, and Jackowski and Rajçhmann state that remissions in this disease correspond to a more or less complete disappearance of the reaction.

The effect of mercurial treatment in cerebro-spinal syphilis is to abolish the reaction after a varying time; it may again, however, become positive. Treatment by inunction and by injections of mercury is more effectual in this respect than by the mouth. Treatment is also more effective in recent cases of syphilitic infection.

Lastly, the reaction may throw light on the causation of cases not otherwise recognised as syphilitic. Thus Ledermann[9] in twenty-three cases of apoplexy found that in ten with a history of syphilis, six, and in thirteen with no such history, three gave a positive reaction; in twelve cases of aortic aneurysm the reaction was positive in ten; and the same result was obtained in fifty per cent. of cases of arterio-sclerosis, the last, if confirmed by other workers, a suggestive result.

In the action of the syphilitic virus on the central nervous system two very different effects are to be distinguished from the outset. Perhaps the most striking characteristic of the nervous system is the extreme differentiation in function of its various parts. In connection with this differentiation the selective action of poisons of all kinds is a marked feature of toxic influences upon it. This effect of various poisons, *e.g.* that of diphtheria in producing paralysis of accommodation, is too well known to need recapitulation. In studying the effects of the syphilitic virus, such selective action is found to be confined to what are known as para- or meta-syphilitic affections. I hold with those who consider that tabes and general paralysis are the sequels of a previous syphilitic infection. These affections present examples of the selective action of poisons directly or remotely due to syphilis. There are (1) the Argyll-Robertson phenomenon, which does not belong to cerebro-spinal syphilis, but is a feature of parasyphilis; (2) the decay of certain fibres of the posterior or sensory nerve roots in tabes; (3) in general paralysis, the destruction of cortical neurones, etc.; (4) primary optic atrophy; (5) tabo-paralysis.

In cerebro-spinal syphilis, on the other hand, there is no evidence of any such selective action of poisons. Its results are due to the long-recognised anatomical lesions of syphilis in the meninges, vessels, and supporting framework of the central nervous system, and the changes in the nervous elements secondary to these, and produced by interference with their nutrition, and by compression from gummatous exudations and tumours. These anatomical changes are uniform in syphilis. At all stages there are exudations of small round cells, necrosis of some of them probably by the destructive action of spirochætes, endarteritis and organisation later of such fibroblasts as escape destruction into sclerosing tissue.

A feature in the course of syphilis that has hitherto not been explained, but which seems likely to be cleared up by modern investigation, is the occurrence of gummatous or vascular lesions long after the primary infection. After long years of apparent freedom a person may develop tertiary symptoms. Once infected, the patient may be liable to syphilitic manifestations during the rest of his life. Further, the syphilitic process may modify or impress its specific characters upon the reaction of the tissues to any subsequent injury. This question is especially important in syphilitic nervous diseases, because they so frequently occur at long intervals after primary infection.

The late occurrence of vascular lesions is not difficult to understand, for from its known action on the arteries syphilis should produce a slowly-advancing change in them, which would lead after a considerable interval of time to narrowing and ultimate thrombosis, and hence to hemiplegia or paraplegia. The mode of production of late gummata is more obscure, but an explanation may be found in the discovery of spirochætes in tertiary lesions. This has been a matter of considerable difficulty, and for some time they were not found, but in a few instances they have now been demonstrated in gummatous tumours, and in the lesions of syphilitic aortitis. Moreover, though tertiary lesions are very rarely infective, Hoffmann succeeded in inoculating an ape from a gumma in a man $3\frac{1}{2}$ years after infection. Hence it is possible that the spirochæte may remain long latent in the tissues and yet be potent for mischief.

It is a common clinical experience that a gummatous lesion may arise at the seat of a blow or other injury. Mott[10] suggests as possible explanations other than the above—from which it would be necessary to suppose that the organisms must be widespread in the tissues for a chance injury to rouse them into local activity, of which evidence is lacking, and which seems, perhaps, inherently improbable—that the spirochætes have remained in some other tissue, *e.g.* the bones or spleen, and escaping thence, act by a kind of metastasis, or that there are forms of trypanosomes having a special affinity for particular tissues, of which there is a certain amount of evidence in the known instances of several cases of general paralysis following syphilitic infection from the same female; or again, the hypothesis above mentioned, that syphilitic infection so modifies tissue changes in the affected person that any inflammatory process shows itself in the peculiar form of the syphilitic lesion.

Passing on now to the clinical features of cerebro-spinal syphilis, we find that they depend on several pathological processes, which it is often difficult to differentiate from each other at the bedside. Briefly, they comprise disease of the vessels and the results of this, new formations or gummata, and inflammations of a specific or gummatous character, especially in the meninges. The symptoms of cerebro-spinal syphilis are not so often produced by one of these processes only as by two or more occurring together in varying combination and relative intensity.

It is convenient to divide the cases into cerebral and spinal, and in some the disease so obviously affects either the brain or cord almost exclusively that this distinction is justified. However, even when such cases, in which symptoms referable either to brain or to cord dominated the clinical aspect, come to pathological investigation it is often found that all parts of the central nervous system are in some degree involved. Bearing in mind this fact, and also the difficulties of differential diagnosis between the several pathological processes, cases of cerebral syphilis may be further divided into those with (1) vascular lesions, (2) gummata, (3) meningitis only, and (4) those with vascular and meningeal lesions or gummata in combination. Especially in the last group both brain and spinal cord are affected together, and there is often a clinical combination at once suggestive of syphilis, a triplegia, a paralysis of one arm and both legs. Taking cases of triplegia first, the history of many is that the patient has had an attack of hemiplegia, due to syphilitic endarteritis, from which recovery has been more or less complete; and then after a variable interval, in the majority about two to three years, but sometimes much longer, even twenty years, paraplegia develops; or more commonly the hemiplegic leg has never completely recovered, and this disability increases together with the development of spastic paralysis in the leg hitherto unaffected. The type of paraplegia in these cases is generally spastic, with much rigidity and exaggeration of reflexes, but little affection of sensation. No doubt this condition may be due to a patch of thrombotic softening in the cord, or to a localised meningitis, but in some patients the signs are suggestive of a slowly-spreading pyramidal tract degeneration. In view of the observations of Dr. Gordon Holmes and others, referred to later, the latter mode of origin, however, is open to question. The prognosis in these cases is bad; I have only once seen a good recovery.

In considering cerebral syphilis, the space at my disposal will only allow me to refer to special points. In cases of cerebro-spinal syphilis some change in the mental processes is generally present, and too much stress can hardly be laid upon the diagnostic value of this. It is especially important amongst the prodromal signs, the patients often being apathetic, drowsy, forgetful, neglectful of work or duty, irritable or passionate. Of all prodromal signs the most frequent is headache, generally with nocturnal exacerbations. Of great importance are recurring paralyses, which are more frequently due to syphilis, especially in patients under forty-five years of age, than to any other cause. The occurrence of transitory aphasia, transient or permanent cranial nerve paralyses, and epileptiform fits in an adult not previously subject to epilepsy, should always suggest an inquiry as to previous syphilitic infection.

1. *In cerebral vascular syphilitic lesions* the early recognition of the nature of prodromal symptoms, amongst which recurring paralysis may be especially mentioned, is particularly important, in order that treatment may be instituted before the affected vessel is completely occluded ; for, as has been often pointed out, once the vessel is closed, and necrosis in the area supplied by it has occurred, the result is the same, to whatever cause, syphilitic or other, the closure of the vessel is due. The common consequence of this form of cerebral syphilis is hemiplegia, with or without aphasia ; syphilitic endarteritis in the retina is not uncommon, and is a valuable aid in diagnosis.

The most common cause of the hemiplegia is thrombosis. Dr. Head considers that hemorrhage is more frequent in cerebral syphilis than is generally held. The difficulty of distinction is, however, great. The final occlusion by a clot of a partly-obliterated vessel may be sudden, and before the final catastrophe the symptoms may not have been sufficient to bring the patient to the doctor. In two of the forty-eight cases of cerebral syphilis on which these remarks are based a diagnosis of hemorrhage appeared legitimate. The onset was apoplectiform with deep coma, and the resulting lesion was a hemiplegia of the ordinary type.

In the diagnosis of such cases from those of simple apoplexy the Wassermann test, the investigation of the cerebro-spinal fluid for lymphocytosis and a globulin reaction are of value. Although in such cases the prognosis as to recovery would hardly differ from that of simple apoplexy, prompt institution of anti-syphilitic treatment is important to prevent extension of the disease to other vessels.

Of what value these reactions may be the following instance shows.

A coachman, aged 37, was admitted with the history that seven days before he was taken ill with a high temperature and aching pains in the limbs ; at the same time he spoke oddly, and left out words in talking. Two days later he became completely aphasic, and lost the use of his right arm. Seven months before this he had been thrown out in a carriage accident and cut his head ; shortly afterwards he had total paralysis of the right third nerve, which passed off after some time.

On admission the right arm, right side of face, and right half of tongue were paralysed. The deep reflexes were exaggerated, and the abdominal absent on the right side. The pupils were unequal, but reacted to light and accommodation. He could only say a few monosyllables, used " yes " and " no " indifferently in reply to questions, had difficulty in repeating words said to him, and also in understanding simple sentences, *e.g.* he did not always correctly carry out simple movements when ordered to do so. He did not speak spontaneously. He could read aloud to a small extent, short words only, and then often substituted wrong ones, and did not understand what he was reading. Under these circumstances it was impossible to get an accurate medical history from him, but so far as could be understood he positively denied any venereal affection. Examination of the cerebro-spinal fluid, however, showed the presence of a decided lymphocytosis, and Noguchi's test was positive.

On this account active treatment with mercurial inunctions and iodide of potassium was begun without delay. He made rapid improvement : the arm began to recover first, then the speech very slowly, but it was three weeks before he began to attempt spontaneous speech.

In the sequel he was able to return home in six weeks' time, having recovered the use of his arm. He was able to write a letter, could read the newspaper and understand what he read, would reply to questions correctly, and to a certain extent spontaneous speech, though imperfect, had now returned. Before he left he told us that he had contracted syphilis at the age of twenty. The prompt institution of treatment was no doubt largely incidental to his recovery.

2. *Cerebral gummata.* As a gumma generally grows into the brain from the meninges it will first invade the cortex, and it is often therefore associated with Jacksonian and sometimes with general epileptiform attacks. It is rarely possible to diagnose a gumma as an isolated lesion, and *post-mortem* cases generally show concomitant meningitis or vascular disease. In some instances, however, clinical symptoms point to a gumma as an isolated lesion, and pathological investigation confirms this. Such gummata may occur in the substance of the cerebrum, cerebellum, or, as in one of my cases, in the medulla oblongata. The signs will then be those of a cerebral tumour ; optic neuritis is not invariable, but is generally present, and is sometimes intense. Should the gumma be in a position to cause hemiplegia, this will be like that produced by other tumours, gradual in incidence, and of longer duration than that produced by vascular lesions.

Perhaps this is the place to put in a word against the administration of large doses of potassium iodide indiscriminately in all cases of intra-cranial tumour on the chance of their being syphilitic. Now that the tests mentioned above are available for diagnosis, such treatment is, to my mind, neither scientific nor justifiable. The condition of the patient deteriorates under the treatment, and valuable time is lost in deciding on the only real means of relief—operation—which all recent experience shows must be carried out early if it is to be successful in cases of cerebral tumours. A possible exception is, if the diagnosis can be made, that of inoperable sarcoma, in which, as in sarcoma of other parts of the body, large doses of potassium iodide may diminish the rate of growth of the tumour, or even check it for a time, but even here the best procedure would be a decompression operation.

In three of my cases it was possible to make the diagnosis of an isolated gumma. One of them died two days after admission, and the sole lesion was a large gumma of old standing in the left frontal lobe, which, if the patient had not been moribund on admission, could have been removed by operation. The other two made a very fair recovery under mercurial inunctions.

3. In cerebral *meningitis* there is a well-marked division of cases according to the affection of the base, convexity of the brain, or the membranes generally. In the two latter forms mental symptoms are as a rule present, and in about half the cases are pronounced, forming a marked clinical feature. The prognosis as to recovery has not seemed to me to be so good as in basic meningitis. In gummatous basic meningitis paralysis of the cranial nerves is the characteristic feature ; the subacute ophthalmoplegias belong here, and paralysis of one side of the tongue, palate and one vocal cord is a well-known combination.

The tongue is sometimes affected alone, showing hemiatrophy. When the disease affects the convexity, tenderness of the skull, either localised or general, is often present, headache is most intense and persistent, and there may be syphilitic disease of the bones of the cranial vault. In the following case an operation was undertaken for the relief of pain and of blindness, due to optic neuritis, and it illustrates the difficulties likely to be met with, and the advantages to be obtained by operation, and also the improbability of a permanent cure.

Annie P., aged 38, single. Previous to 1904 she always enjoyed good health. No history of any syphilitic manifestation could be obtained. In 1904 she began to suffer from nocturnal attacks of intense headache and vomiting of cerebral type. These continued through 1905, and she was in the Hospital twice on that account. On the second occasion there was a large tender node on the skull above the right occipital protuberance, which disappeared almost entirely under treatment with mercury and iodide of potassium. One day early in 1905 she was suddenly taken with loss of vision and vertigo. Subsequently she had frequent attacks separately of (1) loss of vision for one to two minutes ; (2) vertigo, in which there was twitching of the left arm and leg muscles, but never loss of consciousness. At first she had diplopia, lasting a few weeks, and passing off. Her vision steadily deteriorated, first in the right eye. She was found by Mr. Walker to have intense optic neuritis. All these symptoms diminished for a time under specific treatment, but early in 1906 returned with greater intensity, and vision deteriorated in spite of active treatment, until on March 16th, 1906, she had barely perception of light. There had been again for some time a tender bony swelling, 2¼ inches above and in front of the right external occipital protuberance. She was dull and drowsy, with marked slow cerebration, and the optic neuritis had persisted or increased. Under these circumstances I asked Mr. Firth to trephine over the bony swelling, and on doing so he exposed a large patch of carious bone. Trephining was a matter of great difficulty, as the bone was 3¼ inches thick, and dense, like ivory ; a large area was eventually removed. The dura mater was adherent to the bone, and on incising it a diffuse granulation-like tissue was found invading the cortex, occupying the whole area exposed, and extending forwards under the cranium. As much as possible was removed, and the wound closed. The patient made a good recovery. The effect of the operation was seen immediately. The headache and vomiting, previously persistent, ceased at once ; the following day she recognised her brother, and could see the pictures on the wall, and in a week could tell the time by her watch. In a month's time she was able to return home, resume her house-work, go out alone, and to write but not read. She remained well until August, 1907—fifteen months—when the sight began to fail, and the headaches and vertiginous attacks to return. Vision was reduced to a small chink, due to secondary atrophy resulting from the neuritis. She gradually became almost entirely blind from this cause, with return of cerebral symptoms, and appearance of left hemiplegia, and I believe died about two years later.

The occurrence of meningitis soon after primary infection is of considerable importance. In one of my cases meningitis (brain), with marked mental symptoms and slight optic neuritis, came on in a man, aged 26, five months after the infection. In another the symptoms were spinal, and were unmistakably those of spinal meningitis ; they appeared three months after the primary chancre. Mott[11] in opening the discussion on "Syphilis of the Nervous System" at the Neurological Section, gave two examples of this early meningitis, and quoted a case reported by Goldflam in which symptoms of meningitis appeared one month after the chancre, and also one observed by Boidin and Weil, in which infection took place in June and signs of meningitis appeared in August, and four days later the roseolous rash came out. Lumbar puncture showed a pure lymphocytosis, and other observers have noted a lymphocytosis in the cerebro-spinal fluid during the secondary period. Mott suggests that the modern conception of the roseolar rash as due to the escape of spirochætes from the cutaneous capillaries makes it possible that a similar affection of the meninges may occur, and further that in rare instances the spirochætes in the blood may affect the meninges before the skin. Both my cases made a speedy and complete recovery under mercurial inunction, and the same result was obtained in the other cases mentioned above.

With regard to the group of cases in which the signs indicate co-existent vascular and meningeal lesions (gummatous meningitis), like the cases of triplegia mentioned above, evidence of vascular disease in the shape of an attack of hemiplegia not infrequently precedes the more general symptoms by two or three years. Apart from these preceding attacks of hemiplegia the onset is gradual in all. Optic neuritis is present in a few, and syphilitic disease of the retinal vessels is sometimes found, as in the cases of uncomplicated vascular lesions. Symptoms of mental disease are more pronounced than in any other form of cerebral syphilis, and occasionally end in syphilitic dementia, so that the patient has to be removed to an asylum. When mental symptoms are predominant in the case, they do not appear to be associated with predominance of any one form of lesion especially. The prognosis is in my experience bad. No case completely recovered, and improvement only took place to a limited extent.

There may be difficulty in diagnosis from general paralysis, but expansive ideas and delusions are generally absent, the mental changes are associated with various sensory or motor paralyses, hemiplegia, aphasia, cranial nerve palsies, etc. The Argyll-Robertson phenomenon is not present. The cerebro-spinal fluid shows a less-marked lymphocytosis than in general paralyses, and as a rule does not give the Wassermann reaction. Lastly, there is a varying degree of recovery under treatment. However, Turner points out that some cases which apparently begin as cerebral syphilis end as general paresis.

Turning now to some special symptoms of cerebral syphilis, out of forty-five cases *optic neuritis* occurred in nine, and in seven of these there were signs of meningitis with or without other lesions. In four cases, with diffuse cerebral lesions, optic atrophy was found either in one or both eyes. *Epileptiform fits*, indistinguishable from the attacks of general epilepsy occurred in seven cases, in two being associated with localised fits (Jacksonian epilepsy). In two cases fits were the first, and for some time the only, sign of cerebral syphilis, and in three more constituted the most serious and prominent symptom. They were not present in any of my cases of spinal syphilis. The fits were of the major type, one patient only had attacks of *petit mal.* I bring forward these facts because syphilis has been held to be a doubtful cause of epileptic attacks. Recent observations go to show, however, that epileptiform attacks which cannot be clinically distinguished from those of idiopathic epilepsy occur for which no other cause than syphilis can be assigned. Cerebral syphilis is of course a well-known cause of Jacksonian epilepsy.

Dr. Head, [12] in the discussion already alluded to, referred to the occurrence of convulsions and epileptiform attacks in the early stages of syphilis, and Dr. Wilfred Harris mentioned three cases of syphilitic epilepsy arising without any apparent cause, in which there was a strong probability that the lesion was a diffuse meningitis. Certainly in epilepsy starting in adult life syphilis should be remembered as a possible cause, and in case of doubt a Wassermann's test carried out. Unfortunately the results of treatment are often disappointing, the fits tending to persist

I have dealt with the question of the occurrence of the Argyll-Robertson phenomenon elsewhere, [13] and shall not say more than that in its fully-developed form, not a mere sluggish reaction of the pupils, I do not believe that it occurs in cerebro-spinal syphilis, but that its presence is an indication of a further degenerative change in the central nervous system, that is, of parasyphilis. Thus in any case in which it is found it must arouse fear of tabes or general paralysis, or possibly of some other degenerative (parasyphilitic) affection in the future, if signs of such disease are not already manifest. In sixty-seven cases of cerebral and spinal syphilis this sign was present in four cases, of which one developed tabes later, a second was possibly a general paralytic, the third had Erb's syphilitic spastic paraplegia, and in the fourth the sign present at first cleared up under treatment. In six the reaction to light was distinctly present but sluggish, and in three of these also improved by treatment.

A few further points may be added as to the prognosis in cerebral syphilis. In purely vascular lesions, even if treated early, the prognosis is not good, and as we have seen, hemiplegia of vascular origin may be followed a few years later by diffuse vascular and meningeal lesions; hence the very great importance of thorough treatment, and of seeing that the patient returns for fresh courses of treatment, although keeping well, at suitable intervals. In cases of isolated gumma and of pure meningitis the prognosis is fairly good, and there seems less tendency to a recurrence of fresh lesions. Rumpf considers that if the cranial vault is affected the result is likely to be unfavourable. Naunyn's[14] tables state that there is no material difference in percentage of cured and uncured cases, whether considered from the point of view of age at onset of symptoms, or whether they occur within a short interval or over ten years after infection.

Generally speaking, the ultimate prognosis when the symptoms indicate extensive lesions is not good. Although the immediate results of treatment are often very satisfactory, yet there is a strong tendency to the return of the trouble in the same or other forms of lesion, with the final result of a decided and permanent impairment of the patient's mental and physical capacity. He is no longer the same man in his business or profession, which he now carries on with less energy and ability, or too often is unable to carry on at all. Further, in the majority of cases life is shortened.

### *Syphilitic Affections of the Spinal Cord.*

In syphilitic affections of the spinal cord the symptoms in many cases are not solely confined to it; thus there may be a transitory or permanent paralysis of a cranial nerve, especially of the third nerve, as for instance a temporary diplopia. Other signs indicating a general affection of the nervous system may occur, and perhaps be obscured by the predominance of the spinal symptoms. Thus some change in mentality, such as apathy, forgetfulness, cr some confusion of ideas may be present and form an important factor in the elucidation of an obscure case.

The spinal symptoms themselves have often a somewhat bizarre character, and do not correspond with well-defined disease of the systems of neurones in the cord. The state of the reflexes may be contradictory, perhaps absent on one side or in one segment of a limb and exaggerated on the opposite side or in other segments. The presence of symptoms of mixed origin at different levels of the cord, indicating a wide-spread extent of disease and suggesting a variety in the pathological lesions, is of itself of diagnostic value. This wide extent and variety in the nature of the lesions has been laid stress upon by many observers.

Speaking generally, the chief symptoms are spastic paresis or paralysis with some ataxy, exaggeration of the deep reflexes, often to a marked degree, early affection of the bladder, and root lesions.

Of pure meningitis there was one example in twenty-one cases analysed for this lecture, and that one, which occurred three months after infection, has been described above. A passing mention may be made here of the condition of chronic localised meningitis of the cord with a local collection of the cerebro-spinal fluid, occurring generally in the lower dorsal region, and giving rise to symptoms which resemble those of an extra-medullary tumour. This condition is sometimes the result of syphilis.[15] I have once seen an instance of an isolated gumma of the cord (not included in the present series). It was not diagnosed during life. The patient died of a syphilitic visceral disease. The signs of this rare condition would be those of a tumour of the cord.

The most common condition in cases of spinal syphilis is that generally entitled meningo-myelitis. This term is, however, not a very exact one, for in the majority there seems to be no inflammation of the cord itself, but compression of its circumferential zone from meningeal exudation, and patches of softening due to vascular thrombosis. Out of a large number of pathological observations, Gordon Holmes[16] found only one case in which there was a true myelitis. The usual changes were a diffuse meningitis or thickening of the soft membranes of the cord, in varying degree at different levels, with infiltration of round cells around the vessels. The cord itself suffers from compression and interference with its blood supply, but is not actually invaded by the disease. In addition there are peri- and endo-arteritis of the meningeal and intra-spinal vessels, and local patches of softening arise from thrombosis of them.

The tracts in the cord most affected were the spino-cerebellar, direct cerebellar, and Gowers's tract, hence the ataxy that so often accompanies syphilitic spastic paraplegia: the dorsal columns escape to a remarkable degree. He also emphasises the fact that such changes generally occur throughout the nervous system, and are not confined to the cord.

In my experience the onset is acute, rarely sudden, or gradual in about equal numbers. Dr. Head thinks that the prognosis is less favourable in cases of sudden onset, but so far as my cases go the result bore no distinct relation to the mode of onset. Incontinence of urine, and often of fæces, is a common and early symptom. Recovery to the extent of ability to return to full work occurs in only a small proportion; in the rest it is partial only, which generally means that some degree of spastic paresis, with defective control over the bladder, remains.

The disturbances of sensation are, speaking generally, less than those of motion, and recovery from them earlier and more complete. Sensation of any kind is most often not completely lost, but only partially affected. I have found loss to heat and cold most marked and most frequent, next loss to pain (pin-prick), whilst tactile sensation, though often impaired, was not so completely affected. Signs pointing to a lesion of a particular nerve-root or roots are not infrequent, and are valuable in the differential diagnosis. The variable condition of the reflexes, and want of correspondence in the alterations on the two sides or in one limb, have been already alluded to as a diagnostic feature. Thus one knee-jerk may be normal and the other exaggerated, both limbs being paralysed or paretic, or the knee-jerks may be normal with double ankle-clonus, an extensor plantar reflex in one foot and flexor in the other.

The appended charts illustrate some of the sensory changes met with, and in the description the state of the reflexes is also given.

An incomplete or modified Brown-Séquard syndrome may be present, and this also occurs in the rare cases of an isolated gumma in the substance of the cord. In this incomplete form this syndrome is more often found in syphilitic than in any

Fig. 1.

Fig. 2.

Fig. 3.

Fig. 4.

### EXPLANATION OF FIGURES.

In the figures the distribution of sensory loss to light touch is indicated by vertical shading; to pain (pin-prick) by horizontal shading; to heat and cold by oblique shading.

The depth of shading roughly indicates degree of sensory loss.

Fig. 1.—Paraplegia. Diffuse meningitis. In addition there was loss of muscular sense. Muscles of legs were wasted, with much diminished electrical reactions. Incontinence. Right knee-jerk normal, left absent. Superficial reflexes absent.

Fig. 2.—The same case after two months' treatment, when a very considerable degree of motor power had been recovered. Oblique shading indicates loss of sensation to cold only.

Fig. 3.—Paraplegia: sensory loss of moderate degree Root-lesion at 11th to 12th dorsal. Right knee-jerk normal, left exaggerated. Right ankle-jerk normal, left ankle-clonus. Right plantar flexor, left extensor. Loss of muscular sense (partial) in left leg only.

Fig. 4.—Paraplegia. Hyperæsthesia. Zone of hyperæsthesia at level of 11th dorsal, as shaded, and on areas shaded on legs and feet. Legs spasmodically contracted and drawn up on abdomen. Knee-jerks absent. Double ankle-clonus.

other spinal-cord disease, but in its fully-developed form is not often seen. Dr. Wilfred Harris points out that in these cases the crossed analgesia does not rise to nearly so high a level upon the body as the motor palsy.

As in all these cases, thorough and especially early treatment is all-important as regards recovery, it is essential to recognise the initial symptoms. The first is generally pain, either in the back or legs, or often in the distribution of one or more nerve-roots, or a girdle-pain which, as a root-symptom, persists throughout the course of some cases. Then signs of what is now often called "intermittent claudication" may precede the paralysis, that is to say transitory attacks of paresis or even paralysis of the legs, brought on by exertion and disappearing again on resting. Transient paræsthesiæ in the legs or slight loss of control over the bladder are also important, and lastly may be mentioned again the not infrequent coincidence of signs of intra-cranial affection. In any case a lumbar puncture should be made, the spinal fluid examined for lymphocytosis and for globulin, and in case of doubt Wassermann's or Fleming's test carried out. These tests will also be valuable where the multiple and widely-spread lesions of cerebro-spinal syphilis give rise to symptoms which may occasionally simulate those of disseminated sclerosis, although if due attention be paid to the distinctive signs present of either disease there should be no difficulty in diagnosis.

Sometimes the symptoms set up by a chronic syphilitic spinal meningitis may suggest the presence of tabes dorsalis. This is of interest, because of the view held by some observers that the origin of the tabetic lesions is in a meningitis at the point of entry of the posterior roots into the cord. So far as I am able to judge this view is not borne out by the pathological data. In a chronic syphilitic meningitis, however, the Argyll-Robertson pupil is absent and there is the variability in the state of the deep reflexes already discussed. Examination of the cerebro-spinal fluid would show more pronounced lymphocytosis in tabes, and possibly a positive reaction of the fluid to Wassermann's test. Further points given by Mott are the subacute onset, more rapid development, and earlier appearance of the disease after the primary affection in syphilitic meningitis than in tabes.

There is a group of cases of spinal syphilis, clinically well defined, which was first differentiated by Erb in 1892, and is therefore often called Erb's syphilitic spinal paralysis. It is a very chronic affection, and in the cases I have seen developed late, from twelve to twenty years, after the primary infection. In some also of the cases of triplegia, in which paraplegia is slowly engrafted on to a previous hemiplegia, the disease is of this type. The onset is gradual, and the symptoms are, in brief, a spastic paraplegia or paresis combined in some with a certain amount of ataxy, often with hypotony, greatly exaggerated deep reflexes and extensor plantar reflex, sometimes pain in the back and legs or girdle-sensation, loss of sensation often absent and in all cases slight, and early involvement of the bladder. The great exaggeration of the deep reflexes with ankle and knee clonus, out of proportion to the degree of paralysis, is characteristic, as is also the combination of hypotonus with a spastic gait. The disease is slowly progressive, but sometimes remains stationary at a certain stage. Prognosis as to recovery is not good.

The pathology of these cases is somewhat unsettled. Probably the pathological lesion underlying the apparent clinical uniformity is not always the same. Erb concluded that the disease is due to a combined system affection of the lateral and posterior columns with or without a transverse lesion in the dorsal cord. A point at issue is whether a pure degeneration limited to the pyramidal tracts occurs as a consequence of syphilis and accounts for some of these cases. If so, such a degeneration on the motor side would presumably be comparable to that in tabes on the afferent side, and be classed amongst the "parasyphilitic" affections.

Harris[17] quotes Wimmer as stating that such a pure degeneration of the pyramidal tracts has only been found in four cases of those pathologically investigated, most of them presenting a combined system sclerosis. The number of autopsies recorded is, however, few. He also points out, and Williamson [18] records an actual observation in point, that since the peripheral white matter of the cord derives its vascular supply from the short arteries running in from the pia, a gummatous meningitis, by obliterating the nutrient vessels, would cause sclerosis of the periphery of the cord, and hence involve the tracts. Further, ascending and descending degenerations may start from a small patch of softening in the cord, or, as in a case of Dr. Gordon Holmes, in the pons. Nonne, [19] however, in an analysis of cases, gives, in addition to these causes, a primary degeneration of the pyramidal tracts alone, or combined with the postero-lateral tracts. It would therefore seem probable that a certain number of the cases do depend on a pure pyramidal tract degeneration. The only one of my cases of spinal syphilis which presented the Argyll-Robertson sign belonged to the group of Erb's syphilitic spinal paralysis. Wilfred Harris also states that the sign is sometimes met with in these cases. I suggest that it is in the small sub-division of this group, that depends on a pure degeneration of tracts only, and comes under the category of parasyphilitic diseases, that this sign is present.

There are rare cases of spinal syphilis in which atrophy of groups of muscles in the limbs occurs without any sign of sensory affection, and which therefore present the characters of subacute or chronic anterior poliomyelitis.

Some years ago I saw a man who had such a gradual atrophy of the small muscles of the right hand, without pain or sensory change. This recurred twice with an interval of about a year, getting well each time under mercurial and electrical treatment. Two years later he returned again with the same affection, but this time no recovery took place, and after he had attended some time as an out-patient I lost sight of him. In another case of a man aged 35, with cerebral syphilis, left hemiplegia and epileptiform fits, there was wasting of the small muscles of the left hand, and of some of those of the forearm, of gradual onset. The affected muscles gave the reaction of degeneration, but showed no fibrillary tremor. Except for some pain in the limb sensation was unaffected. A certain degree of recovery ensued under treatment. In a third case, a man aged 26, with left hemiplegia and paraplegia, wasting of the muscles of the left leg below the knee came on in the course of ten days, and was attended with considerable pain in the limb. When seen there was marked wasting of the muscles, with diminished reaction to both the faradic and constant currents. The only affection of sensation was some hyperæsthesia in the distribution of the left external popliteal nerve.

Spiller has published a case of acute anterior poliomyelitis due to thrombosis of branches of the anterior spinal artery, which showed marked thickening of their coats. He emphasises the importance of thrombosis of vessels as the cause of paralysis of acute onset in spinal syphilis. Hoffmann[20] shows that acute and chronic anterior poliomyelitis may occur in the course of syphilis, and reports a case in which a man aged 20, with signs of congenital syphilis, was so affected. Harris[21] describes three cases of syphilitic poliomyelitis, occurring very late after the primary infection, in which complete or partial Argyll-Robertson pupil was present, and in one case loss of most of the deep reflexes. In the diagnosis from progressive muscular atrophy he lays stress upon the more acute onset and presence of R. D. Examination of the cerebro-spinal fluid showed no lymphocytosis or excess of albumin.

In hereditary syphilis the cord is occasionally affected, apart from the occurrence of tabes, and such affection may either show itself in childhood, or for the first time in an adolescent who has previously been in good health. The changes in the cord in new-born infants, the subjects of congenital syphilis, have been recently described by Toyofuku,[22] and closely correspond to those found by Ranke in the brain in congenital syphilitic infants. The most striking change is a thickening and lymphocytic infiltration of the pia-arachnoid, surrounding the cord in its whole extent, and contrasting with the very slight and occasional affection of the dura mater. Equally marked is a similar infiltration of the cord itself throughout its whole transverse section. "Rod cells" were also found in addition to lymphocytes and plasma cells. Vascular disease was slight and not characteristic. The changes in the cord itself stood in no relation to those in the pia mater. The ganglion cells mostly stained normally.

In two cases of congenital syphilis under my observation, one, in whom the signs of disease first appeared at 19 years of age, showed a universal swelling and infiltration of the soft membranes of the cord.

A. A., aged 21, tram conductor. Father, mother, four brothers and two sisters in good health, one brother and one sister died in infancy. Good health until aged 19. In October, 1901, he was admitted to Hospital for pains in back of head and neck of some weeks' duration, with frequent attacks of sickness, worse after food. The pain was intermittent; there was also failure of sight and weakness of legs.

On admission he complained of pains in the head, was sick and very drowsy, being roused with difficulty. Opacity of cornea, from old interstitial keratitis. Pupils dilated, acted to light and accommodation. Paresis of right external rectus. Nystagmus on looking to right. Double optic neuritis – swelling +2D. Hutchinson's teeth. Paresis left side of face. No paralysis of limbs. Right knee-jerk exaggerated, otherwise reflexes all normal. Plantar flexor. He had a fit in which left arm was convulsed.

After six weeks' treatment with mercurial inunctions and potassium iodide he went out well, except that he was a weakly youth with feeble musculature. Returned to work for some weeks, and was then laid up again, and had alternate periods of work and illness during this year, doing about four months' work altogether.

On February 8th, 1902, symptoms of weakness in the legs, headache and sickness came on again. He stayed in bed, getting worse, but managed to walk up to Hospital with great difficulty on February 12th. He was ordered to come in, but there being no bed vacant he returned home. On the 13th he lost all power in his legs, could not get out of bed, and had retention of urine; during the night the urine dribbled away. On the 14th he could not even turn in bed, and the bowels were obstinately constipated. On the 16th he was admitted, and appeared very ill, dull and heavy, cheeks hollow and sunken.

There was absolute loss of movement in lower limbs, intercostals and abdominal muscles moved in respiration. Loss of sensation below fourth dorsal segment, as shown in chart; it varied in degree. No upper zone of hyperæsthesia; arms unaffected. Bladder over-distended. No tenderness or irregularity in spines of vertebræ, no stiffness in back.

There was constant priapism. Optic discs showed remains of old optic neuritis. Cranial nerves unaffected. Knee-jerks exaggerated, Babinski's sign present on both sides. Since admission incontinence of fæces. Mental state dull and drowsy, answered questions correctly but slowly. Some pain complained of between the shoulders.

Fig. 5.

Fig. 5.—Paraplegia in hereditary syphilis. Male, aged 20. Loss of sensation indicated by shading as in other figures. In addition the vibrations of a tuning-fork were not felt over the vertebral spines below the 5th dorsal, nor over bony prominences of pelvis and lower extremities.

February 27th.—The patient has had several fits, in which he lost consciousness for a few minutes. The face and upper, but not lower limbs were convulsed, the left arm being especially affected. Legs absolutely anæsthetic to light touch, but pin-prick is felt in places over them.

The fits were now preceded by a cry, and they were seen to begin in the left side of the face and left arm, and then to spread to the right arm.

During March and April he was drowsy and apathetic, the legs were absolutely powerless; the reflexes much exaggerated, with double ankle-clonus and Babinski's sign; retention of urine and incontinence of fæces continued; there was priapism; upper abdominal reflexes present, lower and cremasteric absent. The condition of sensation is given in Fig. V. A pin-prick was felt as low as the fifth intercostal space, but localised a space too low; posteriorly, not felt below sixth dorsal spine; deep pressure and the tuning-fork vibrations were also not felt below this, and the latter not recognised on the bones of the pelvis and lower extremities.

In view of the sharp demarcation of upper limit of anæsthesia, and of the order of onset of symptoms—(1) increase of deep reflexes, (2) loss of motor power, (3) loss of sensation—it seemed probable, though I was well aware of the possibility of an extensive diffuse meningitis with secondary changes in the cord, that there might be a gummatous tumour or a localised collection of fluid pressing upon the cord in the middle or lower dorsal region, and that this might be relieved by operation. In view also of the facts that after a full trial of medical treatment the condition was getting worse, and that if unrelieved the patient was condemned to hopeless paralysis, with the prospect of death at no very distant period, it was decided to do a laminectomy. The cord was therefore exposed by the removal of the upper dorsal laminæ and spines. The dura mater appeared healthy, and on opening it a diffuse internal meningitis was found, and within this the cord itself was soft and hyperæmic. The condition was evidently too extensive to be dealt with by operation, which the patient bore well, and from which he made a good recovery. Except for the disappearance of ankle-clonus, no improvement resulted. He died about four weeks afterwards from the natural consequences of the disease.

At the *post-mortem* examination the pia-arachnoid was thickened and opaque at the base and over the cerebellum. The cerebral ventricles and iter were much distended, and full of clear fluid. The spinal dura mater was externally normal. On slitting it up the pia-arachnoid was thickened, gummy or jelly-like—adherent to the cord and also to the dura—over the cervical and lumbar enlargements, and over the most part of the dorsal cord. This change was most marked over the cervical enlargement, and here and over the lumbar one there was much injection of vessels, and the cord was softened.

The second case was a boy aged 10, who showed marked signs of infantilism, and in appearance looked like a little old man. He was emaciated. He could only say a few simple words when admitted, and his mental condition was so dull as not to be far removed from imbecility. He kept himself clean, and had no loss of control over the bladder. He could neither stand nor walk. The legs were drawn up on the abdomen, and the muscles were small and wasted from disuse. There was no sensory affection ; the reflexes were exaggerated. He had an enormous congenital syphilitic liver, smooth, hard, and with a sharp edge. It reached the umbilicus, and the left lobe was especially enlarged to form a prominent rounded tumour in the epigastric region. So much did it resemble a tumour that an exploratory operation was done. A small piece of the liver removed at this operation showed on microscopical examination the typical histological structure of congenital syphilitic liver. Under a long course of mercurial treatment with K.I. and massage and passive movements to the lower limbs, he made a remarkable recovery as regards the paraplegia. At first he walked with a very ataxic feeble gait, but contiued to improve steadily until he was able to walk and run about like a normal boy. His mental state showed a considerable degree of improvement, and he learnt to talk well, but would be still classed as feeble-minded. The liver also greatly diminished in size, and his nutrition became normal.

The discussion of syphilitic disease of the brain and cord, dealt with even as inadequately as I fear it has been done, has left me no time for the consideration of the parasyphilitic diseases—tabes and general paralysis—further than to say that I agree with those who hold that they are a sequel of previous syphilitic infection.

Finally, a few words may be added as to treatment, in which the most important points are rest in bed and the prompt and efficient administration of mercury. Of course, patients in an acute or sub-acute stage, or disabled by some form of paralysis, are physically unable to get about ; but complete rest is equally necessary and beneficial, although the need for it may not be so immediately obvious in cases of chronic disease during the first period of treatment. For carrying out mercurial treatment, I have used inunction, which personally I have not found difficult. Care must be taken that it is efficiently done. After about two months of daily inunctions, special care being taken of the mouth, etc., an interval is left, but it is then most important that the patient should return in about three months' time for a further course, and again at intervals of about six months for about three years. In a few of my later cases intramuscular injections of perchloride of mercury were employed. Iodide of potassium can be given in addition, especially in the later stages, in gradually-increasing doses ; but though it may be administered for long periods with advantage, I do not think it is advisable to let the patient take it continuously; nor when it is necessary to push it, and it is well known that in some cases large doses are required to produce effect, have I found that any advantage is to be gained by going beyond a dose of 30 or 40 grains every four hours.

### REFERENCES.

[1] Mott, Morison Lectures, *Brit. M. J.*, 1909, i. 454.

[2] For reasons why they are not so found, *vide* Mott, *loc. cit.*

[3] Morison Lectures, *loc. cit.*

[4] Mott, "The Comparative Neuropathology of Trypanosome and Spirochæte Infections," *Proc. Roy. Soc. Med.* (Pathological Section), 1910, iv. 1.

[5] Quoted by Mott, Morison Lectures, *loc. cit.*

[6] *Lancet,*, 1909 i. 1512.

[7] Quoted by Mott, *loc. cit.*

[8] *Jahresb. d. Neurol. u Psychiat.*, 1910, xiii. 428.

[9] *Neurol. Centralbl.*, 1910, xxix. 1229.

[10] Loc. cit., *Brit. M. J.*, 1909, i. 457.

[11] *Proc. Roy. Soc. Med.* (Neurological Section), 1909-10, iii. 36.

[12] *Proc. Roy. Soc Med.*, Neurol. Sect., 1909–10, iii. 49.

[13] *Brit. M. J.*, 1903, ii. 1634, and *Ibid.*, 1910, i. 296.

[14] Quoted by Turner, Allbutt and Rolleston's *System of Medicine*, 1910, viii. 344.

[15] For a full account *vide* Sir V. Horsley, *Brit. M. J.*, 1909, i. 513.

[16] Discussion on Syphilis, *Proc. Roy. Soc. Med.* (Neurological Section), 1910, iii. 60.

[17] Allbutt and Rolleston's *System of Medicine*, 1910, vii. 727.

[18] *Diseases of the Spinal Cord*, 1908, p. 393.

[19] Quoted by Williamson, *loc. cit.*

[20] *Jahresb. d. Neurol. u. Psychiat.*, 1910, xiii. 433.

[21] *Loc. cit.*, p. 731.

[22] *Jahresb. d. Neurol. u. Psychiat.*, 1910, xiii. 433.

## Figures and Tables

**Fig. 1. Fig. 2. Fig. 3. Fig. 4. f1:**
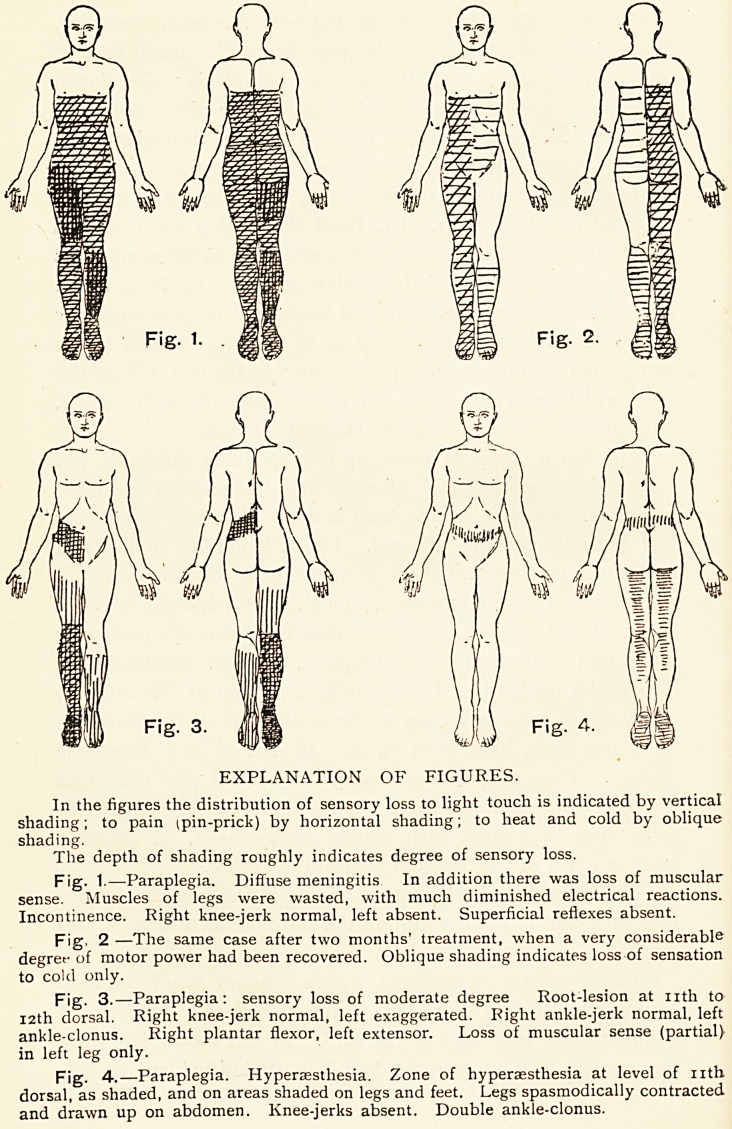


**Fig. 5. f2:**